# A novel alkali and thermotolerant protease from *Aeromonas* spp. retrieved from wastewater

**DOI:** 10.1038/s41598-024-76004-w

**Published:** 2024-10-29

**Authors:** Najmeh Sodagar, Razieh Jalal, Mohsen Fathi Najafi, Ahmad Reza Bahrami

**Affiliations:** 1 Department of Chemistry, Faculty of Science, Ferdowsi University of Mashhad, Mashhad, Iran; 2 Novel Diagnostics and Therapeutics Research Group, Institute of Biotechnology, Ferdowsi University of Mashhad, Mashhad, Iran; 3 Industrial Biotechnology Research Group, Institute of Biotechnology, Ferdowsi University of Mashhad, Mashhad, Iran; 4 Department of Pathobiology, Faculty of Veterinary Medicine, Ferdowsi University of Mashhad, Mashhad, Iran; 5 Department of Biology, Faculty of Science, Ferdowsi University of Mashhad, Mashhad, Iran

**Keywords:** *Aeromonas* spp., Alkaline serine protease, Thermal stability, Wastewater, SDS resistance., Proteases, Industrial microbiology

## Abstract

Enzymes are integral to numerous industrial processes, with a growing global demand for various enzyme types. Protease enzymes, in particular, have proven to be cost-effective, stable, and compatible alternatives to traditional chemical processes in both industrial and environmental applications. In this study, an alkaline protease-producing strain of *Aeromonas* spp. was isolated from a wastewater treatment plant in Iran. The protease production was confirmed by culturing the strain on casein agar medium. The bacterium was identified through morphological, biochemical, and 16 S rRNA sequencing analyses. The optimal culture medium for bacterial growth and enzyme production was obtained using peptone, salt, yeast extract, galactose, and CaCl₂ at an initial pH of 8. Maximum protease production was achieved after 20 h of incubation at 40 °C. To partially purify the enzyme, the supernatant of the bacterial culture medium was first centrifuged, and the enzyme was precipitated using ammonium sulfate, followed by dialysis. Zymography revealed the production of one type of protease during bacterial growth. The partially purified protease exhibited optimal activity at pH 8.5 and maximum stability at pH 9. The optimum temperature for maximum enzyme activity was observed at 50 °C, with 100% residual activity retained for 1 h at 0 °C. The effect of metal ions on enzyme activity was assessed, revealing that KCl induced the most significant effects (*p <* 0.0001) on enzyme activity. Chemical amino acid modifiers and inhibitors, such as EDTA, DEPSI, and IAA, did not exhibit significant inhibition. In contrast, PMSF and HNBB significantly (*p <* 0.0001) reduced enzyme activity, suggesting that the enzyme could be classified as a serine protease. The protease also demonstrated high stability in the presence of 2% SDS, showing no signs inactivation. The alkaline pH optimum, thermal stability, and resistance to SDS exhibited by the protease produced by the *Aeromonas* strain are particularly promising characteristics that warrant further investigation. Based on preliminary tests and the enzyme’s characteristics, this protease can be recommended for various applications, pending further studies.

**Supplementary Information:**

The online version contains supplementary material available at 10.1038/s41598-024-76004-w.

## Introduction

The industrial demand for microbial enzymes is rising, as they offer cost-effective, sustainable, and eco-friendly alternatives to traditional chemical-based processes across various industries, including food, detergent, textile, and biofuel production^1^. The production and optimization of microbial enzymes are more stable and straightforward compared to those derived from animals and plants. Additionally, microorganisms require less space for growth, have shorter growth periods, and their enzymes function effectively under a wide range of physical and chemical conditions. Therefore, microbial enzymes are considered the best sources of enzymes due to their stability, simplicity, and versatility^2–4^. Industrial effluents from leather, poultry, and dairy industries, which can be highly saline, alkaline, or contain toxic chemicals, are significant sources of mesophilic and extremophilic microbes^5,6^. Most proteases produced by microbes are extracellular and secreted into the culture medium^7^. Bacteria and fungi are the most common microorganisms that produce these enzymes. There are two main methods for producing microbial alkaline proteases: solid-state fermentation and submerged fermentation. Several factors influence the production of alkaline proteases, including temperature, pH, and the presence of enzyme inhibitors^6^. Microbial proteases account for approximately 70% of all industrial enzymes and are extensively utilized across various industries^3^. Alkaline proteases are active in a neutral to alkaline pH range. Among proteases, alkaline proteases are particularly significant, accounting for nearly 60% of total enzyme sales worldwide^8^. They have garnered interest due to their extensive use in various industrial processes, including photography, wastewater treatment, detergent manufacturing, peptide synthesis, and leather processing^9,10^. The substrates used for bacterial growth in enzyme production are costly, accounting for 30–40% of the final production costs. Therefore, further investigation is necessary to develop cost-effective culture media^11^. Many industrial processes operate at elevated temperatures, which can denature and destabilize many enzymes^12^. Consequently, there is significant interest in discovering new thermostable proteases that can withstand the harsh conditions of industrial processes^13^. Conducting industrial processes at higher temperatures also reduces the risk of contamination by mesophilic bacteria^14^. *Bacillus*, *Halomonas*, *Serratia*, *Arthrobacter*, *Pseudomonas*, and *Alcaligenes* are among the most important bacteria for alkaline protease production. Optimizing their growth conditions is essential for enhancing enzyme yield from these microorganisms^15,16^. The substantial industrial and commercial significance of high-tolerance proteases necessitates the continuous search for novel microbial sources capable of producing these enzymes. In this context, we isolated an alkali-thermotolerant, protease-producing bacterium from wastewater. Additionally, we optimized protease production and activity under various physicochemical conditions, including pH, temperature, and compatibility with SDS, to evaluate its potential for industrial applications.

## Materials and methods

### Isolation and screening of protease-producing bacteria

Wastewater samples were collected through roughing filtration from two wastewater treatment plants, Kenvist and Charmshahr, in Mashhad, Iran. The screening of bacterial isolates was performed at both primary and secondary levels. For the primary screening, wastewater samples were spread on casein agar plates containing the following components per liter: peptone 2.5 g, NaCl 2.0 g, casein 10.0 g, yeast extract 2.0 g, glucose 5.0 g, and bacteriological agar 15.0 g (with the pH adjusted to 7). The plates were then incubated at 37 °C for 24 h. Following the primary screening, a secondary screening was conducted using the same method to analyze the formation of clearing zones. The diameters of the clearing zones and bacterial colonies were measured in two dimensions. The clearing zone index (CI) for protease activity was calculated using the following formula^17^:

$$\:\text{C}\text{l}\text{e}\text{a}\text{r}\text{i}\text{n}\text{g}\:\text{Z}\text{o}\text{n}\text{e}\:\text{I}\text{n}\text{d}\text{e}\text{x}\:\left(\text{C}\text{I}\right)=\frac{\text{H}\text{a}\text{l}\text{o}\:\text{z}\text{o}\text{n}\text{e}\:\text{d}\text{i}\text{a}\text{m}\text{e}\text{t}\text{e}\text{r}+\text{C}\text{o}\text{l}\text{o}\text{n}\text{y}\:\text{d}\text{i}\text{a}\text{m}\text{e}\text{t}\text{e}\text{r}}{\text{C}\text{o}\text{l}\text{o}\text{n}\text{y}\:\text{d}\text{i}\text{a}\text{m}\text{e}\text{t}\text{e}\text{r}}$$

The isolate with the highest zone index was selected for further studies.

### Protease production

Protease production by the isolate was conducted in Tryptone Soya Broth (TSB) medium (pH 7.0) containing the following components per liter: casein peptone 17.0 g, soya peptone 3.0 g, sodium chloride 5.0 g, dipotassium hydrogen phosphate 2.5 g, and glucose 2.5 g^18^. The cultivation was performed in an orbital shaking incubator at 37 °C and 180 rpm for 72 h. After incubation, the bacterial culture was centrifuged at 4 °C and 2,650 g for 10 min. The cell-free supernatant was then collected and assayed for protease activity.

### Protease activity assay

Protease activity was measured according to the method of Kunitz, with slight modifications^19^. Briefly, 450 µL of 50 mM Tris-HCl (pH 8.0) containing 1% (w/v) casein and 100 µL of the enzyme solution were mixed and incubated at 37 °C for 30 min. The reaction was terminated by adding 1 mL of trichloroacetic acid (TCA) solution [5% (w/v) TCA, 9% (w/v) sodium-acetate, and 9% (v/v) acetic acid]. The mixture was then left at room temperature for 30 min, and the precipitate was removed by centrifugation at 15,000 g for 10 min. Finally, the absorbance of the supernatant was measured at 280 nm using a UV spectrophotometer.

Unit Definition: one unit of enzyme activity is defined as the amount of enzyme that causes an increase of 0.001 units in optical density (O.D) at 280 nm under the specific conditions.

### Determination of protein content

The total protein content was estimated using bovine serum albumin (BSA) as the standard protein, according to the Bradford method^20^. Briefly, a series of BSA standard solutions (0–1 mg/mL) were prepared to construct a calibration curve. Coomassie Brilliant Blue G-250 dye reagent (1 mL) was mixed with both the blank and protein samples (10 µL each) in separate test tubes. The mixtures were then pipetted and incubated for 15 min at room temperature. The absorbance was measured at 595 nm in triplicates.

### Identification of the most protease-producible isolate

#### Morphological and biochemical characterization

The bacterium was identified using morphological and biochemical characteristics according to Bergey’s Manual of Systematic Bacteriology^21^. Macroscopic features, including the color and shape of the colony, were observed, and microscopic analyses, such as Gram staining and endospore staining, were performed using the Schaeffer–Fulton method^22^.

#### Molecular characterization

The sequencing of the 16 S rRNA gene was conducted to identify the isolated bacterial strain. Briefly, single colonies were inoculated in nutrient broth and incubated at 37 °C for 16 h. Subsequently, 1 mL of the culture was centrifuged at 4 °C for 10 min at a speed of 10,000 g. The extraction of nucleic acid was performed following the guidelines provided with the bacterial DNA isolation kit (Denazist Asia, Iran). DNA purity was assessed by measuring the absorbance ratio at 260 nm and 280 nm. For the amplification of the 16 S rRNA gene, oligonucleotide primers Eub_1_ forward (5´-AGAGTTTGATCCTGGCTC-3´) and Eub_2_ reverse (5´-GCTCGTTGCGGGACTTAACC-3´) were utilized. The polymerase chain reaction (PCR) thermal conditions were set as follows: an initial temperature of 98 ℃ for 30 s. This was followed by 38 cycles, each consisting of 98 ℃ for 30 s, 56 ℃ for 30 s, and 70 ℃ for 30 s; with a final extension at 72 °C for 7 min. The PCR products were visualized on a 0.8% agarose gel stained with ethidium bromide. A 1Kb DNA Ladder (Sinaclon, Iran) was used as a standard marker. The 16 S rRNA gene of the bacterial isolate was sequenced in both directions by Microsynth, Switzerland. Analysis and assembly of the sequencing data were performed using SnapGene software. The homology of the 16 S rRNA sequence was examined using BLASTn, comparing it against sequences in the National Center for Biotechnology Information (NCBI) database (http://www.ncbi.nlm.nih.gov). A phylogenetic tree was generated using the neighbor-joining (NJ) method via MEGA-X software (version 10.0.5).

### Optimization of culture conditions for protease production

The OFAT (one-factor-at-a-time) method was employed to investigate the effects of nitrogen, carbon, and mineral sources, as well as pH, temperature, and time course, on bacterial growth and protease production. Initially, seven nitrogen sources (ammonium sulfate, sodium nitrate, soy peptone, yeast extract, meat extract, casein, and casein peptone; 0.5% w/v), four carbon sources (sucrose, galactose, and fructose at a concentration of 0.5% w/v), a combination of glucose (0.5%) and lactose (0.2%), and five mineral sources (NaCl, KCl, CaCl_2_, MgSO_4_, and KH_2_PO_4_, at a concentration of 0.2% w/v) were screened. These were individually added to the basal medium (0.5% glucose, 0.2% NaCl) inoculated with bacteria. Then, pH (4 to 10), temperature (20 to 70 °C), and time course (0 to 72 h) were screened sequentially, using the optimal nitrogen, carbon, and mineral sources^23^. All media were incubated at 37 ℃ with agitation at 180 rpm, and both growth and protease production were monitored. After incubation, the absorbance at 600 nm was measured to assess bacterial growth, and protease activity was estimated using the assay described previously.

### Partial purification of protease

The protease underwent partial purification via ammonium sulfate precipitation^24^. The cell-free supernatant was obtained by centrifuging a 24-hour bacterial culture at 2,650 g for 15 min at 4 °C. Ammonium sulfate was gradually added to the supernatant and incubated at 4 °C for 2 h to achieve 75% saturation. The precipitate was collected by centrifugation at 2,650 g for 10 min at 4 °C. The protein precipitate was resuspended in a minimal volume of 20 mM Tris-HCl buffer (pH 8.5) and dialyzed overnight for mineral removal against 500 mL of the same buffer at 4 °C using a dialysis membrane (Nalo, diameter of 60 mm, molecular weight cut-off approximately 10,000–20,000 Daltons).

### SDS-PAGE

SDS-PAGE was performed according to the Laemmli method to determine the protein profile during the optimization stages^25^. Briefly, a 15 µL aliquot of the sample was added to a 5% stacking gel and a 12% resolving gel, then allowed to run at 80 V for 1.5 to 2 h. The gel was subsequently washed and stained with Coomassie Brilliant Blue G-250 staining solution for 2 to 2.5 h at 25 °C with rotation. Afterwards, it was de-stained in a de-staining solution (2% orthophosphoric acid, 10% ethanol, 88% distilled water) overnight. A 10–250 kDa ladder (Sinaclon, Iran) was used as a standard marker.

### Zymography

Zymography was performed using the overlay technique as described by Walker^26^. This method is useful for the detection and semi-quantitative analysis of proteases in the gel. First, the samples were mixed with a loading buffer, that was prepared without 2-mercaptoethanol and not subjected to heat treatment. The samples were electrophoresed on a 10% polyacrylamide gel, running at 20 mA for 4 h at 4 °C. The Native-PAGE was then sandwiched with an agarose gel containing 1% casein and incubated at 40 °C for 20 min. Finally, the agarose-casein gel was stained with Coomassie Brilliant Blue G-250 to visualize the zones of hydrolysis.

### Characterization of partially purified protease

#### Effect of pH

The effect of pH on protease activity was evaluated using casein substrates prepared in different pH ranges: sodium acetate (pH 4 to 6), sodium phosphate (pH 6 to 8), and Tris-HCl (pH 8 to 10). The mixtures were incubated for 30 min at 37 ℃, after which the activities were measured under standard assay conditions. The pH stability of the protease was assessed by per-incubating the enzyme at different pH values (4 to 10) with using the aforementioned buffers at 4 ℃ for 24 h. The enzyme’s residual activity was then measured at its optimum pH and expressed as relative activity.

#### Effect of temperature

The effect of temperature on protease activity was evaluated by incubating the enzyme at various temperatures (0 to 80 °C) for 30 min at the optimum pH. Thermal stability was studied by pre-incubating the enzyme for 30 min at temperatures ranging from − 20 to 80 °C. Protease activity was measured at the optimum temperature using 1% (w/v) casein as a substrate and was reported as relative activity.

#### Effects of metal ions

The effects of different metal ions at concentrations of 5 and 10 mM, such as Na^+^, K^+^, Ba^2+^, Mg^2+^, Zn^2+^, Ca^2+^, Hg^2+^, CO^2+^, and Pb^2+^, on the relative activity of the enzyme were assessed. Protease activity was measured under standard assay conditions, with the enzyme activity in the absence of any metal ions considered as 100% activity.

#### Effect of inhibitors

The partially purified enzyme was pre-incubated with different concentrations of EDTA (2, 5, 10, 15, 20, and 25 mM) at 4 °C for 24 h. The residual activities were determined with respect to the control.

#### Chemical modification and inhibitor

The partially purified enzyme was pre-incubated with different concentrations of chemical modifiers, namely phenylmethylsulfonyl fluoride (PMSF), iodoacetamide (IAA), 2-hydroxy-5-nitrobenzyl bromide (HNBB), and diethylpyrocarbonate (DEPSI), each at a final concentration of 5 mM, at 4 °C for 24 h. The relative activities were determined with respect to the control.

#### Effect of SDS

The impact of surfactants on protease activity was evaluated using various concentrations of sodium dodecyl sulfate (SDS) at 0.1%, 0.5%, 1%, 1.5%, 1.8%, and 2% (w/v).

### Statistical analysis

The experiments were conducted at least three times, and the results were expressed as the mean ± standard deviation (SD). Statistical analysis was performed using one-way analysis of variance (ANOVA) with GraphPad Prism software (version 10.2.3) at significance levels of *p* < 0.05, < 0.01, < 0.001, and < 0.0001.

## Results and discussion

### Isolation and screening for protease producers

**Fig. 1:**
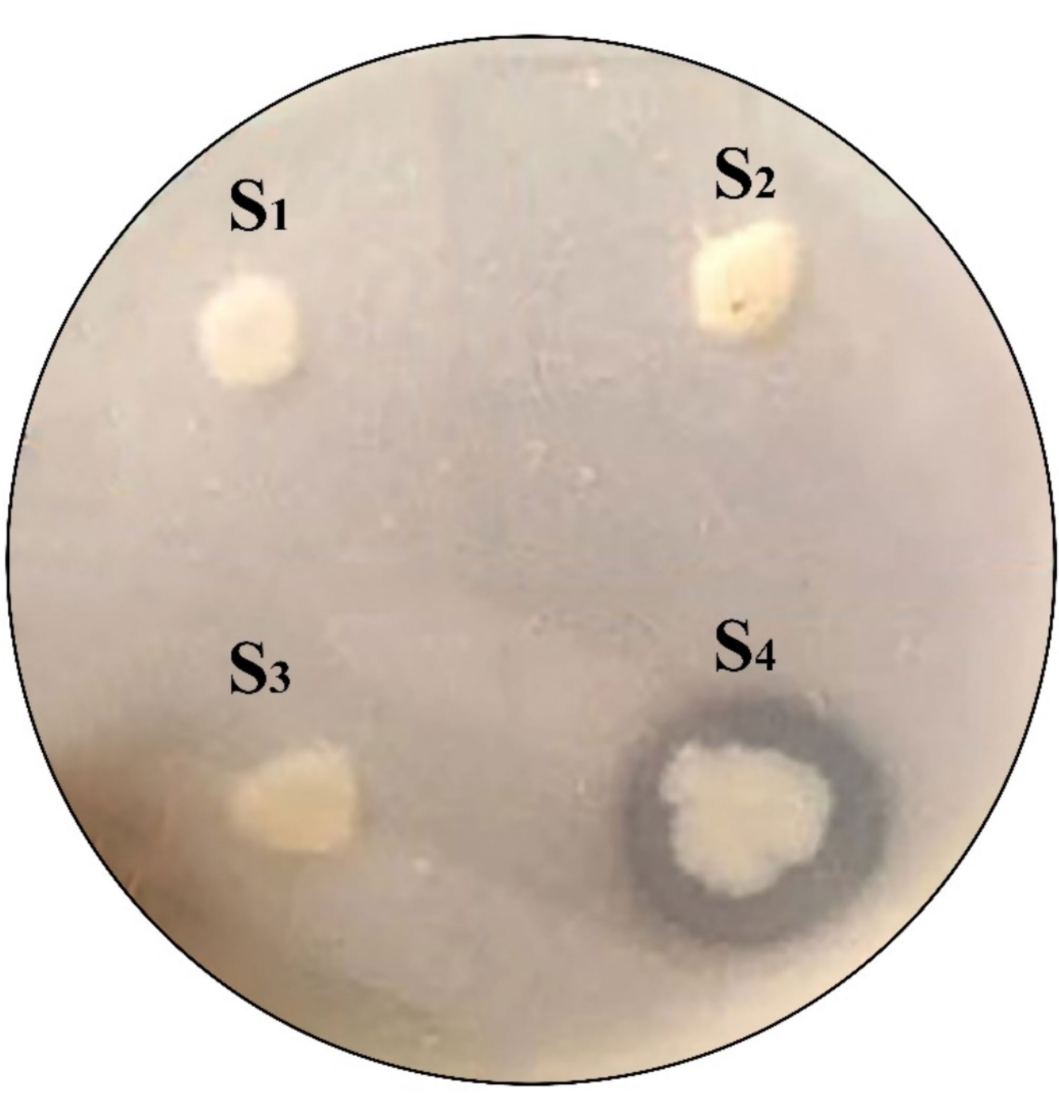
Casein plate assay used to screen bacteria isolated from the wastewater treatment plant: Colony No. S_4_ demonstrated proteolytic activity through the hydrolysis of casein (cropped). The original photo is presented in Supplementary 1, Fig. S1 online.

Among the four colonies (S_1_-S_4_) isolated from the secondary screening of the Kenvist wastewater treatment plant, colony S_4_, exhibited the largest clear zone on a casein agar plate (Fig. 1). Additionally, the clearing zone index (CI) of colony S_4_ was the highest (1.62) among the tested colonies. The CI values for the four colonies are provided in Supplementary 2, Table S1, online. These selected isolates were subsequently compared based on protease activity measurements. The highest mean protease activity, 115.3 U/mL, was recorded for the isolate from colony S_4_ after 24 h of incubation, demonstrating a correlation between the qualitative and quantitative analyses of the studied bacteria. Meanwhile, the colonies isolated from the Charmshahr wastewater treatment plant showed no clear zones.

### Bacterium identification

#### Morphological and biochemical characterization

**Fig. 2:**
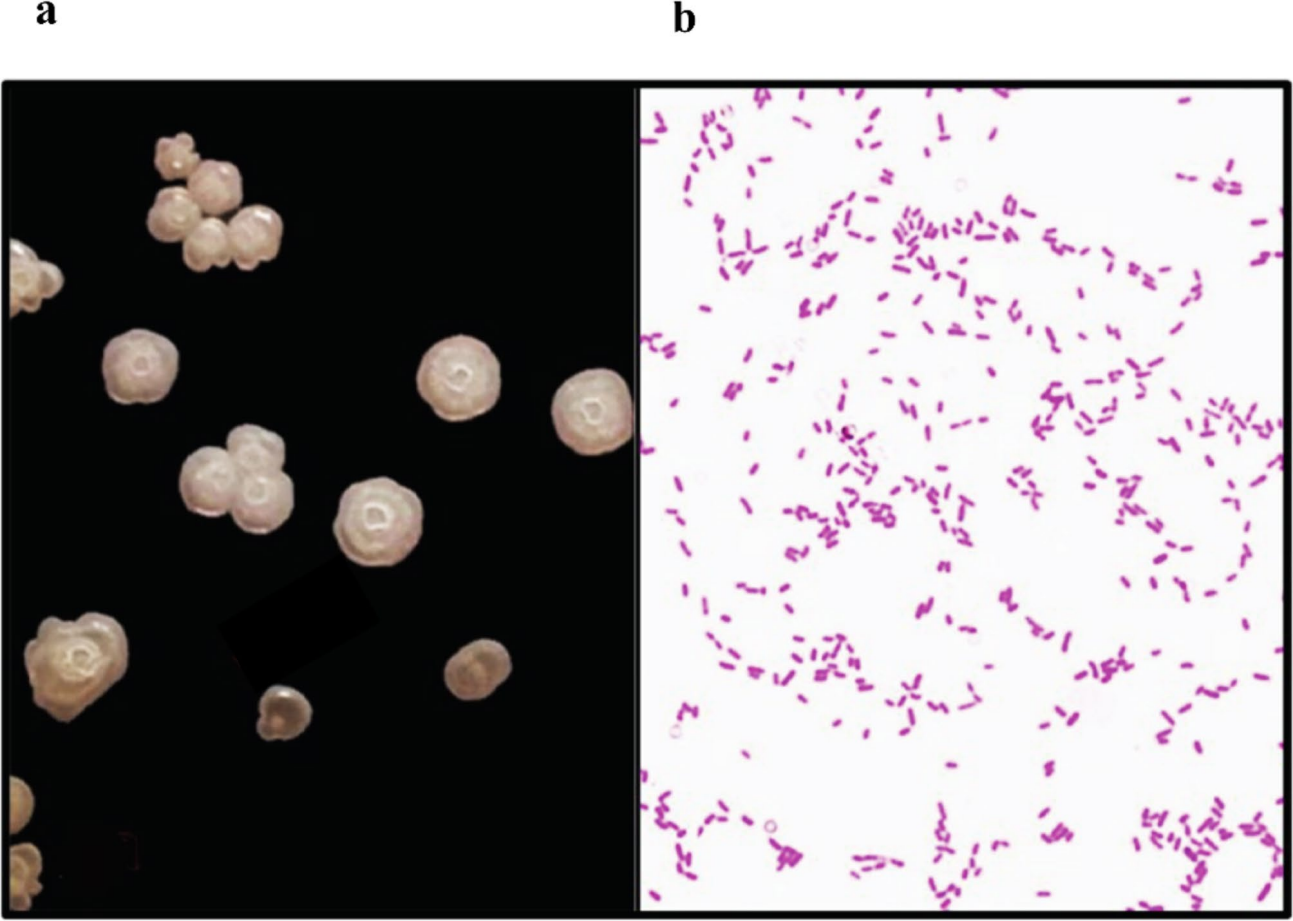
Morphological characteristics of the isolate: Colony morphology of S_4_ observed on nutrient agar medium after incubation at 37 ℃ for 24 h (**a**). Gram staining of the isolate displaying Gram-negative rods (**b**).

The morphological and biochemical characteristics of the isolate are provided in Supplementary 2, Table S2, online. Colony S_4_ exhibited an irregular morphology on the nutrient agar plate (Fig. 2a). Additionally, under Gram staining, the isolate displayed a short rod-shaped morphology (Fig. 2b).

#### Molecular characterization

**Fig. 3:**
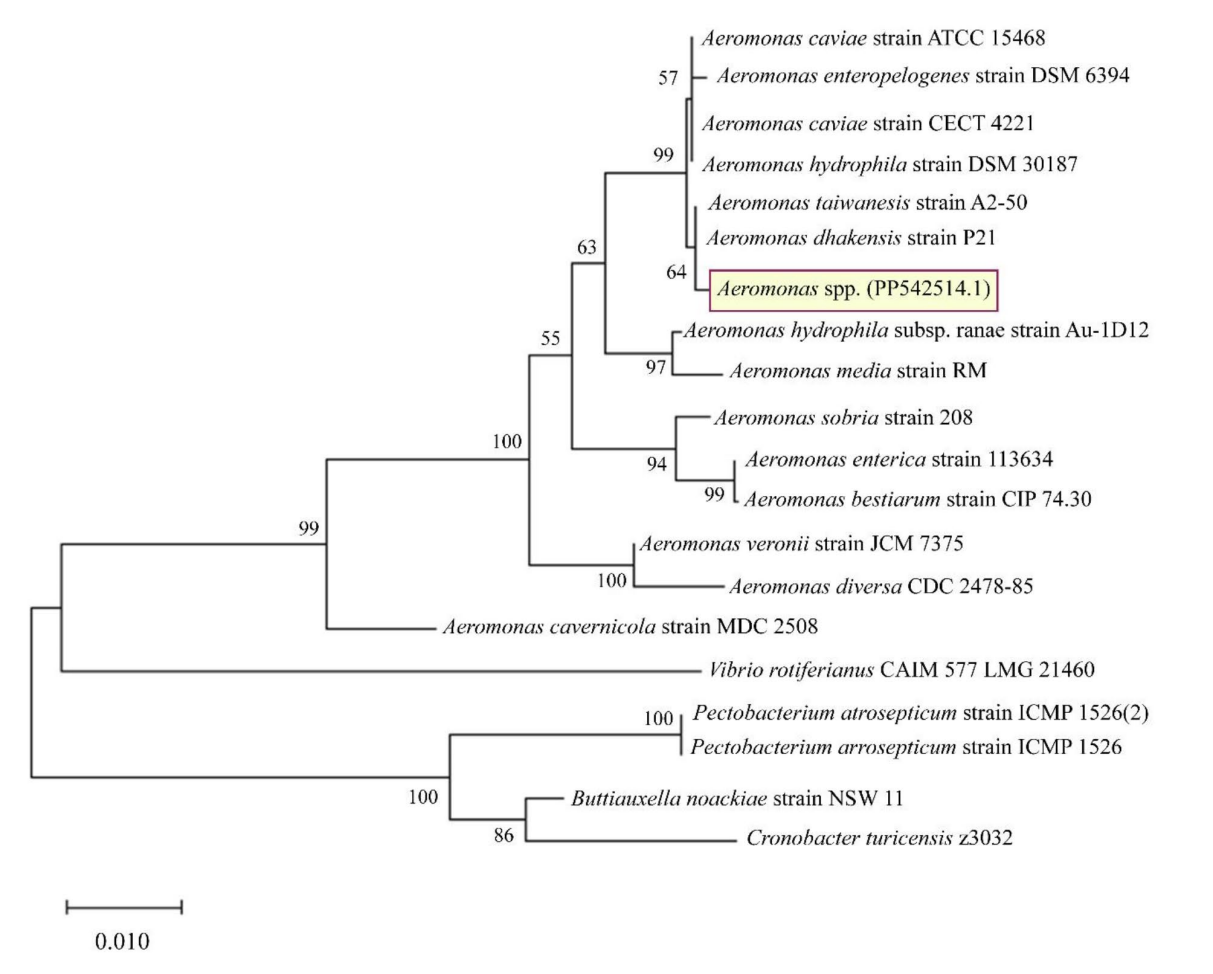
Phylogenetic tree of the bacterium isolated from a wastewater plant, named ‘*Aeromonas* spp.’. The tree was constructed using the Neighbor-Joining method and illustrates the close relationship of the isolate to type strains based on 16 S rRNA gene sequencing data generated by MEGA-X. The numbers at the branch nodes indicate the levels of bootstrap support obtained from 500 replicates.The scale bar represents a phylogenetic distance of 0.010 nucleotide changes per site.

Genomic DNA was extracted followed by the amplification of the 16 S rRNA gene. The gels are shown in Supplementary 2, Fig. S1, online. The primers, which are well-conserved across prokaryotic organisms, have been shown to amplify a partial region of the rRNA gene, measuring 750 bp^13^. The findings of the BLAST analysis demonstrated that the bacterium was classified within the *Aeromonas* genus. The sequence was submitted to NCBI (accession number: PP542514.1). The strain exhibits 99.20%, 99.06%, 99.06%, and 99.07% genetic similarity with *Aeromonas taiwanensis*, *Aeromonas caviae*, *Aeromonas hydrophila*, and *Aeromonas dhakensis*, respectively. Furthermore, the resulting phylogenetic tree indicated a close relationship between *Aeromonas* spp. and both *Aeromonas dhakensis* strain P21 and *Aeromonas taiwanensis* strain A2-50 (Fig. 3). In summary, sequencing of the 16 S rRNA gene enables the identification of genera with up to 90% accuracy and species with a precision range of 65–83%. However, in 14% of instances, the isolates cannot be identified^27–29^. The isolate was identified as *Aeromonas* spp. through a combination of 16 S rRNA analyses, biochemical tests, and morphological characteristics.

The genus *Aeromonas* now belongs to the class Gammaproteobacterias, the order Aeromonadales, and the family *Aeromonadaceae*, which includes three genera: *Aeromonas*, *Oceanimonas*, and *Tolumonas*. Members of this genus are facultative anaerobes that ferment D-glucose into acid, with or without gas production. Furthermore, they are characterized as Gram-negative, rod-shaped, and non-spore-forming bacilli^30^. *Aeromonas* species are catalase- and oxidase-positive, capable of degrading nitrates to nitrites, and produce various kinds of extracellular hydrolytic enzymes such as peptidases, esterase, amylase, elastase, deoxyribonuclease, chitinase, and lipase^31^. *Aeromonas* is commonly found in various natural environments, particularly in aquatic ecosystems^32^.

### Optimization of protease production

#### Effect of nitrogen sources

**Fig. 4:**
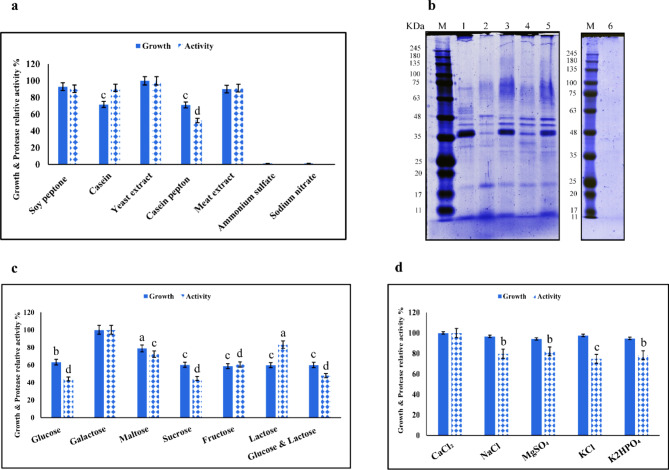
Effect of different nitrogen sources on the growth and protease activity of *Aeromonas* spp., with yeast extract as the control (**a**). SDS-PAGE analysis of the protein profile produced by the bacterium grown in different nitrogen sources (cropped), M: marker, 1: soy peptone, 2: casein, 3: yeast extract, 4: casein peptone, 5: meat extract, 6: ammonium sufate (**b**). Effect of different carbon sources on the growth and protease activity of *Aeromonas* spp., with galactose as the control (**c**). Effect of different mineral sources on the growth and protease activity of *Aeromonas* spp., with CaCl_2_ as the control (**d**). Data are presented as mean ± SD, and (**a**, **b**, **c** and **d**) represent significant differences with the controls at *p* < 0.05, 0.01, 0.001 and 0.0001, respectively. The original gels are presented in Supplementary 1, Figs. S4a and S4b online.

As described earlier, different nitrogen sources were evaluated for their effects on both growth and protease activity. Among the various nitrogen sources tested, a medium supplemented with yeast extract yielded the maximum protease production (135.5 U/mL) for the *Aeromonas* strain between 18 to 20 h of incubation. Figure 4a shows the effect of different nitrogen sources on growth and protease activity after 20 h of incubation. Protease activity was increased by organic nitrogen sources, whereas inorganic nitrogen sources supported neither the growth of *Aeromonas* spp. nor protease production. This finding aligns with the observations presented by Liu and Hsieh (1969), which demonstrated that enzyme synthesis was notably inhibited by ammonium compounds, although the effect on growth was only slight^33^. Figure 4b shows the protein profile produced by the bacterium grown in different nitrogen sources. The quantitative increase in enzyme production observed during the fermentation process may be attributed to the enhanced synthesis or hydrolysis of certain proteins, which is influenced by the nitrogen sources used in the fermentation medium. The choice of nitrogen sources can significantly impact the metabolic pathways and regulatory mechanisms within the microorganisms, ultimately affecting the expression and activity of the target enzyme. Certain nitrogen sources, such as organic nitrogen compounds (e.g., peptones, yeast extracts) or inorganic nitrogen compounds (e.g., ammonium salts), can provide a more favorable environment for microorganisms to synthesize the desired enzyme. Singh et al.. observed the highest protease induction from *Aeromonas hydrophila* LA1 in the presence of casein^34^.

### Effect of carbon sources

The results showed that the maximum protease production was obtained in the medium supplemented with galactose (131.5 U/mL), and the lowest production was observed in the medium containing glucose (58 U/mL), between 18 to 20 h of incubation (Fig. 4c). O’Reilly and Day (1983) investigated the effect of carbon sources on protease production by *Aeromonas hydrophila*, finding that the optimal yield was obtained with media supplemented with sucrose compared to other sources^35^. Enzyme induction by various carbon sources differs among strains within the same species^34^. In general, medium composition, including carbon and nitrogen sources, as well as environmental changes, significantly influence the growth and protease production by microorganisms^36–39^.

### Effect of mineral sources

The impact of different mineral sources on growth and protease production was evaluated using a medium supplemented with galactose and yeast extract as carbon and nitrogen sources, respectively. All tested mineral sources supported growth and protease production; however, protease activity increased by 20% in the presence of calcium chloride compared to the other minerals. Figure 4d displays the impact of various mineral sources on both the growth and protease activity in *Aeromonas* spp. Mokashe et al. (2015) and Adinarayana et al. (2003) reported that Ca^2+^ and Mg^2+^ ions resulted in the maximum enzyme yield^40,41^.

### Effect of initial pH

**Fig. 5:**
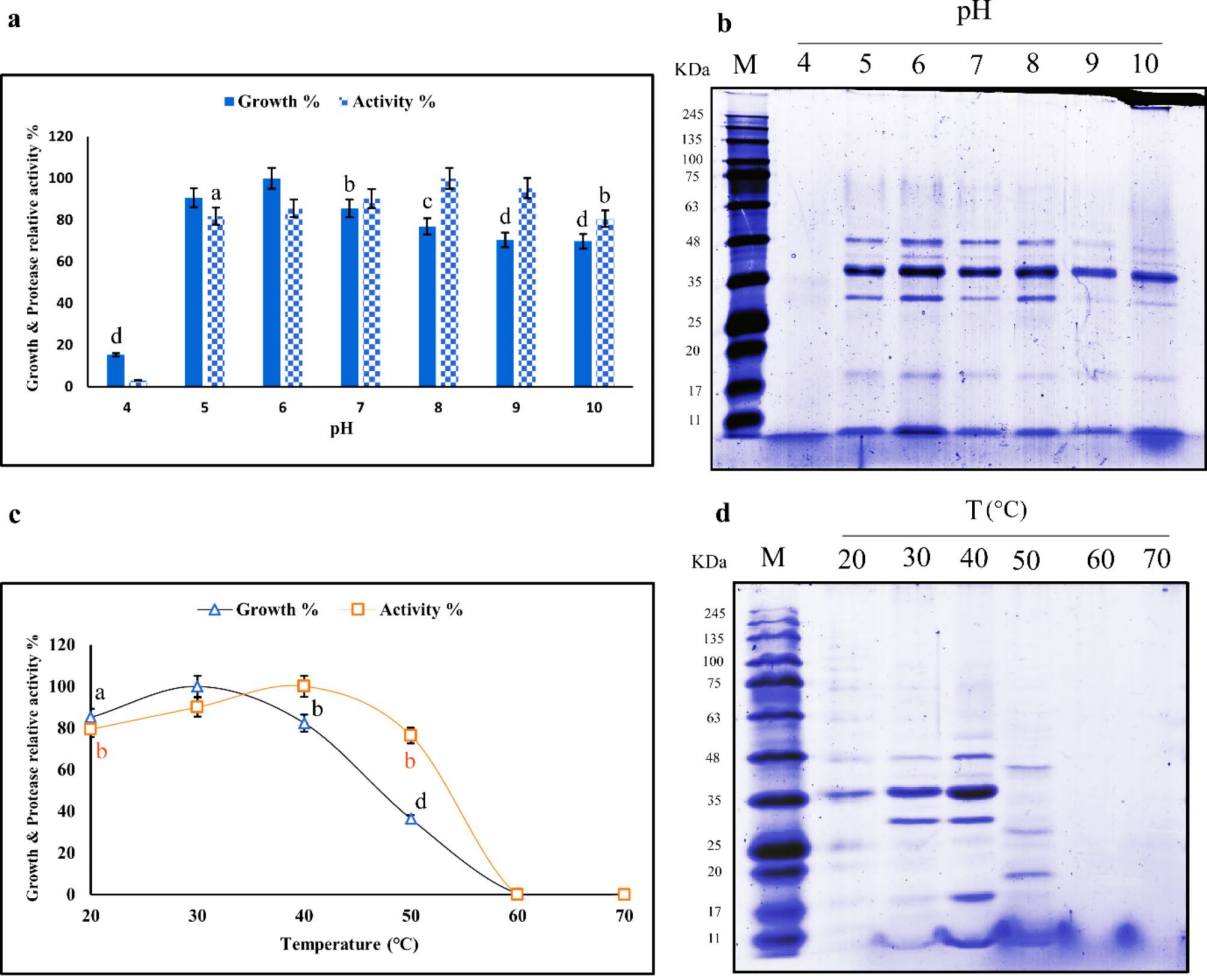
Effect of initial pH on the growth and protease activity of *Aeromonas* spp. The optimal pH for growth and activity were 6 and 8, respectively (**a**). SDS-PAGE analysis of the protein profile produced by the bacterium grown at different initial pH levels (cropped), M: marker (**b**). Effect of temperature on growth and protease activity of *Aeromonas* spp. The optimal temperatures for growth and activity were 30 and 40 ℃, respectively (**c**). SDS-PAGE analysis of the protein profile produced by the bacterium grown at different temperatures (cropped) (**d**). Data are presented as mean ± SD, and (**a**, **b**, **c** and **d**) represent significant differences from the optimum pH and temperature at *p* < 0.05, 0.01, 0.001 and 0.0001, respectively. The original gels are presented in Supplementary 1, Figs. S5 and S6 online.

The organism was able to grow and produce protease over a wide pH range from 5 to 10, with maximal growth occurring at pH 6 and maximal protease production was detected at pH 8 (Fig. 5a). These results highlight the effect of pH and its importance for bacterial growth and protease production^36^. In a similar study conducted on *Bacillus subtilis* spp. subtilis strains, NRRL B-3384, the highest bacterial growth was observed at pH 6, but the maximum protease activity was detected at pH 8^42^. Figure 5b presents the protein profiles resulting from the growth of the bacterium at various initial pH levels, demonstrating fewer protein bands at pH 4 compared to those observed at other pH values. According to Sharma et al. (2017), the enzyme production process and the transport of different components across cell membranes are greatly influenced by the pH of the culture medium^43^. After 24 h of growth in different pH media, all final culture pH levels reached 7.5-8.0. Therefore, it can be concluded that, after growing at any pH, the bacterium adjusts its micro-environment to a suitable pH, revealing the alkaliphilic nature of the *Aeromonas* spp. strain. In another report, Bhattacherjee et al. (2021) observed a maximum yield of protease at pH 6 in *Aeromonas veronii* CMF^44^. According to Ratzke and Gore (2018), the optimum pH varies among different species and even among different strains of the same species originating from various natural environments^45^.

### Effect of temperature

In the present study, the strain of *Aeromonas* exhibited cell growth at temperature ranging from 20 ℃ to 40 ℃, with maximum growth observed at 30 ℃. Similarly, the protease activity produced by this organism was achieved between 30 ℃ and 50 ℃, with maximal activity at 40 ℃ (Fig. 5c). A drastic decrease in growth and protease yield was observed with an increase in temperature, which may be attributed to the denaturation of the protease enzyme caused by elevated temperature and the accumulation of toxic metabolites produced by the bacterium in the culture medium. Additionally, the protein pattern of the protease produced by the bacterium grown at different temperatures was analyzed, revealing no distinct protein bands at 60 ℃ and 70 ℃ (Fig. 5d). Temperature and pH are two important factors that affect protease production in most organisms^44^. In a related study, Bhattacherjee et al.. (2021) reported that protease production by *Aeromonas veronii* CMF was highest at an optimum temperature of 35 °C^44^. The optimal growth temperature for *Aeromonas hydrophila* is reported to be 28 °C^46^, however, it is capable of thriving in a temperature range from 4 °C to 42 °C^47^. Principally, Aeromonads grow optimally within a temperature range of 0 to 45 °C, depending on the species or strain. Mesophilic *Aeromonas* strains thrive at 35 to 37 °C, while psychrophilic *Aeromonas* strains grow well at 22 to 25 °C^32^.

### Growth kinetics of the bacterium and protease production

**Fig. 6:**
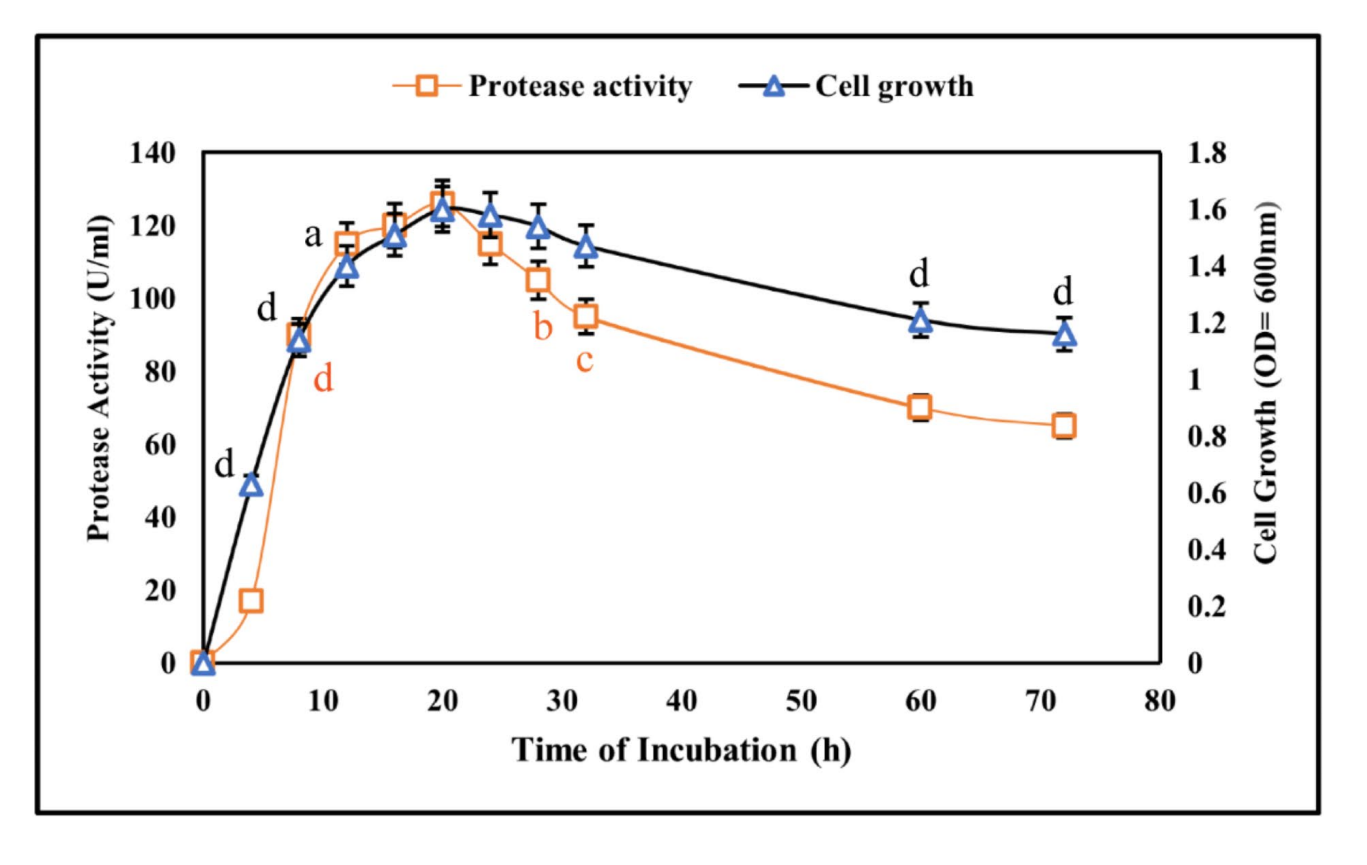
Growth kinetics and protease activity of *Aeromonas* spp. The optimal time for growth and activity was 20 h. Data are presented as mean ± SD, and (**a**, **b**, **c** and **d**) represent significant differences from the optimum time at *p* < 0.05, 0.01, 0.001 and 0.0001, respectively.

The growth pattern of *Aeromonas* spp. and protease production were observed for up to 72 h with interval sample collection (Fig. 6). Growth and protease secretion began at the onset of the exponential phase and reached their maximum levels during the stationary phase. Maximum growth and protease activity were observed between 18 and 20 h of incubation, after which both gradually decreased. This indicates that protease production was proportional to cell growth. Protease activity reached 60% of the initial maximum activity at 72 h. A shorter incubation period reduces production costs and increases profitability on an industrial scale, compared to the additional yield gained with a longer incubation period^48^. Bacteria exhibit maximum protease production in the late logarithmic and early stationary phases when the primary sources of carbon and nitrogen in the culture medium are depleted, necessitating the secretion of more protease to digest complex nutrients^49^. Several factors, including the ratio of carbon to nitrogen, the presence of easily metabolizable sugars, ionic strength, and minerals in the bacterial culture medium, affect the production of alkaline protease. Moreover, parameters such as aeration level, pH, temperature, and incubation time can significantly influence protease production^50–52^. Therefore, optimization the composition of the production medium and the cultivation conditions is necessary for the production of protease, resulting in a higher yield of alkaline protease for commercial purposes^53^.

### Protease partial purification and characterization

As shown in Table 1, the specific activity of the protease increased 1.2-fold and 2.39-fold after the 75% ammonium sulfate precipitation and dialysis steps, respectively, with recoveries of 49.99 and 60.34%, based on the original strength of the protease in the culture supernatant of *Aeromonas* spp.

**Table 1:** Purification steps for the protease produced by A*eromonas* Spp.

Purification stage	Total protein (mg)	Total activity (U)	Specific Activity (U/mg)	Fold	Yield (%)
Culture supernatant	184	11,408	62	1	100
Ammonium sulphate precipitation	76	5702	75.02	1.21	49.99
Dialysis	46.44	6883	148.21	2.39	60.34

### Zymogram analysis

**Fig. 7:**
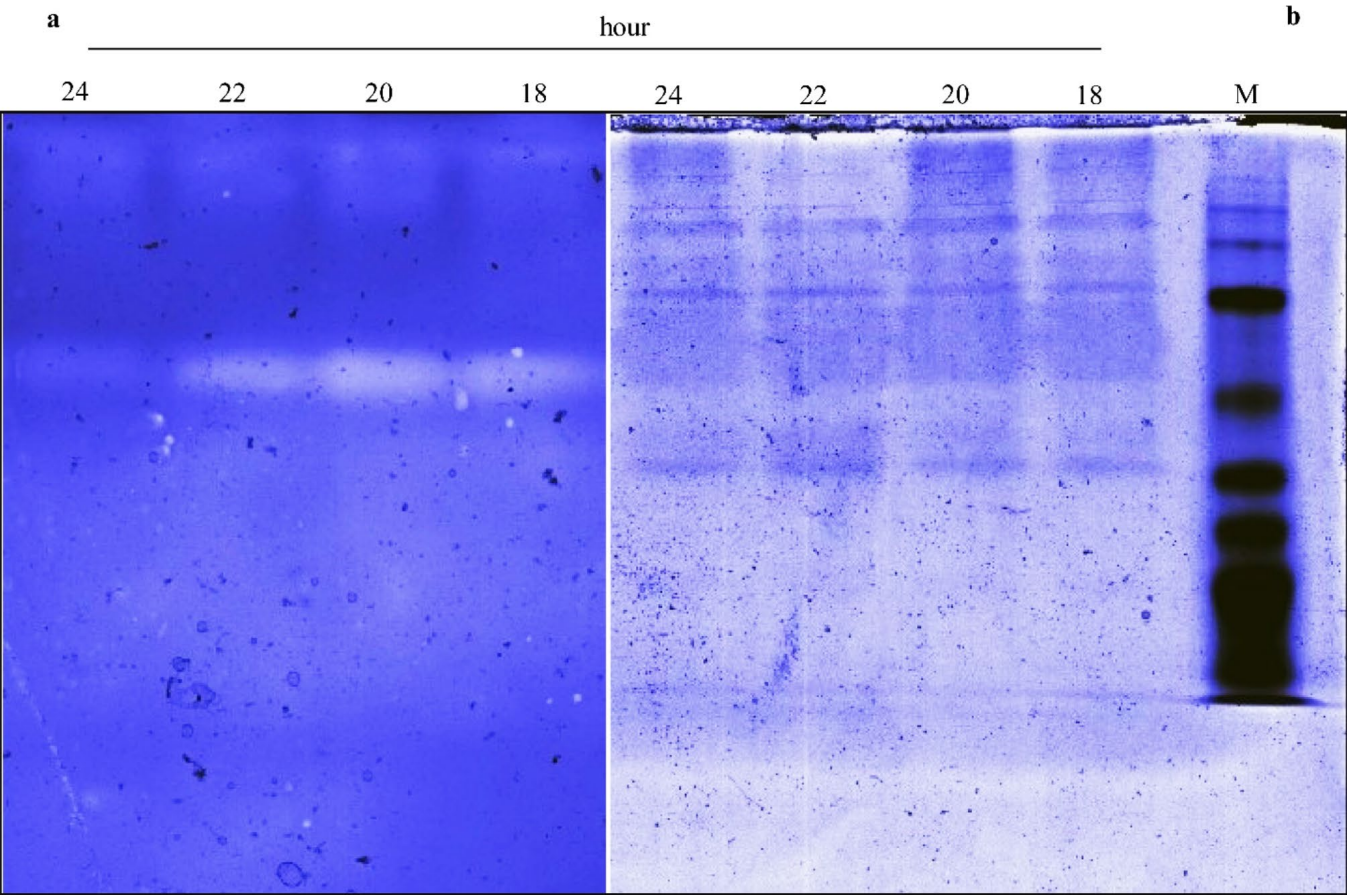
Zymographic analysis of partially purified protease from *Aeromonas* spp. after 18 to 24 h of incubation on 1% agarose gel (cropped) (**a**). 10% native polyacrylamide gel (cropped), M: marker (**b**). The original gels are presented in Supplementary 1, Figs. S7 and S8 online.

The partially purified protease was analyzed by zymography using Native-PAGE. The results, shown in Fig. 7a, suggest that *Aeromonas* spp. produced one type of protease at different incubation times (18 to 24 h). A clear zone of hydrolysis corresponding to an incubation time of 20 h exhibited the highest caseinolytic activity, while at 24 h, it displayed weaker proteolytic activity. This suggests that the bacterium reached its peak production at 20 h of incubation and then gradually decreased. These results are consistent with the data reported in the growth kinetics and protease production experiments. The results for the 10% polyacrylamide gel are shown in Fig. 7b.

### Characterization of partially purified protease

#### Effect of pH on the activity and stability of the protease

**Fig. 8:**
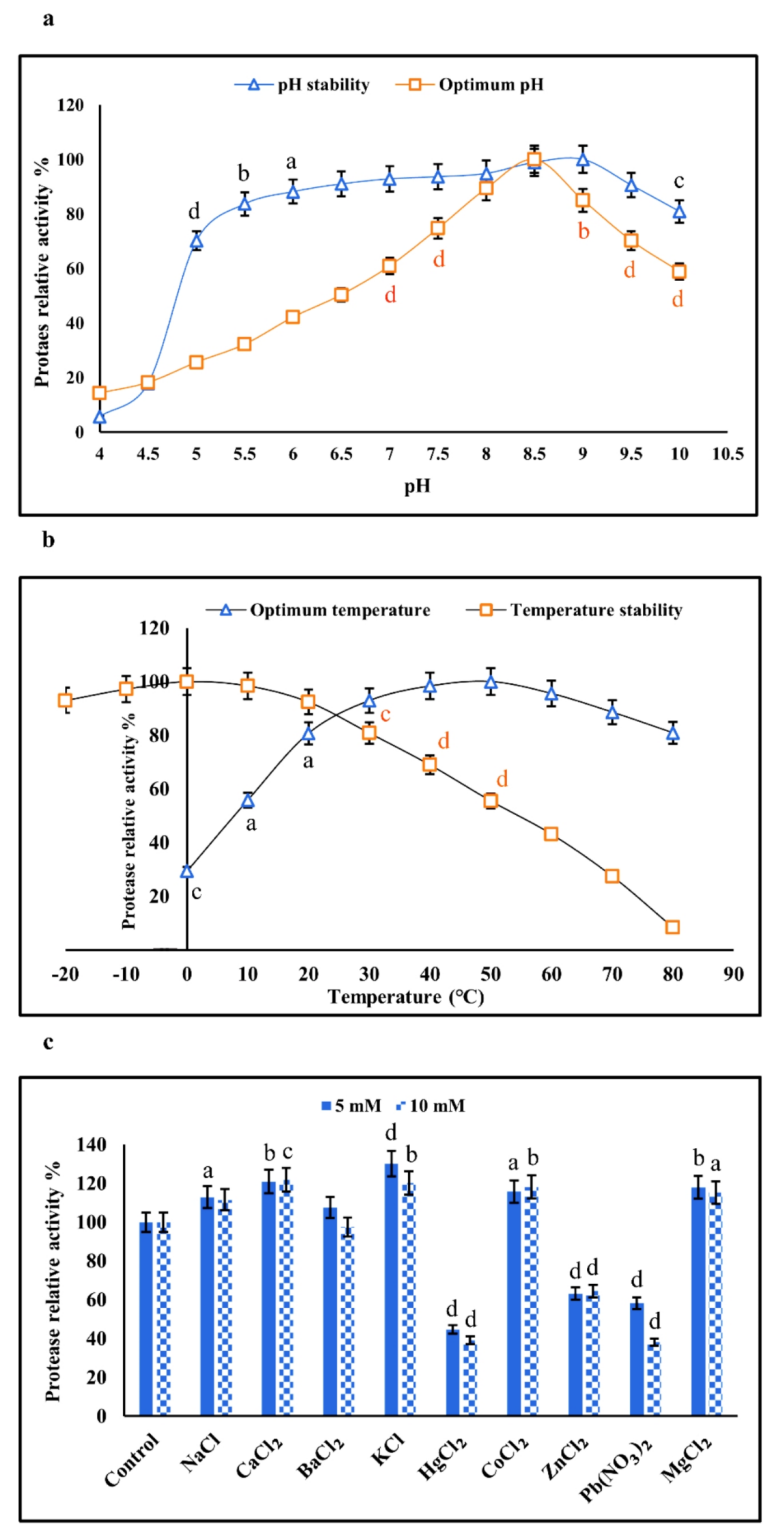
Effect of pH on protease activity and stability. The stability and optimum pH were 8.5 and 9, repectively (**a**). Effect of temperature on protease activity and stability. The stability and optimum temperaures were 0 and 50 ℃, repectively (**b**). Effect of metal ions on protease activity produced by Aeromonas spp. (**c**). Data are presented as mean ± SD, and (a, b, b and d) represent significant differences between groups at p < 0.05, 0.01, 0.001, and 0.0001, respectively.

The protease from *Aeromonas* spp. was active between pH 4 and 10, exhibiting optimal activity at pH 8.5. The enzyme retained nearly 60% of its relative activity at pH 10, indicating high alkaline tolerance. Additionally, the protease demonstrated high stability across pH ranges from 5 to 10, and retained 100% of its stability at an alkaline pH of 9 when incubated at 4 ℃ for 24 h (Fig. 8a). Considering that the protease was very stable over a broad pH range and maintained approximately 80% of its original activity between pH 5 and 10, it can be concluded that the enzyme possesses a highly stable secondary and tertiary structure, likely due to the formation of numerous disulfide bonds. The pH stability of the enzymes is a critical property for industrial use^54,55^. The optimal pH for protease production determined in this study corresponds with the optimum pH for protease from *Aeromonas caviae*^56^ and *Aeromonas hydrophila* PC5^57^. The optimal pH for alkaline proteases typically ranges from 9 to 11^58^. Similarly, results were observed for serine protease from *Aeromonas hydrophila* Ni39 over the pH range of 8 to 10, with maximum activity at pH 9^59^.

#### Effect of temperature on the activity and stability of the protease

The relative activities of the protease at various temperatures, using casein as the substrate, are shown in Fig. 8b. The enzyme activity increased with temperature within the range of 0 ºC to 50 ºC and retained 80% of its activity at 80 °C, indicating its thermotolerant nature. The thermal stability of the protease was studied at 50 °C after per-incubating at various temperatures (from − 20 to 80 °C). As shown in Fig. 8b, the highest thermal stability of the protease was observed at 0 °C, decreasing to 10% at 80 °C. This finding is consistent with those reported for alkaline proteases from *Aeromonas caviae* and *Aeromonas caviae* AP34, where proteases activity was lost after incubation at 80 °C^60,61^. Laishram (2016) also demonstrated the highest protease activity from *Aeromonas caviae* at 50 °C^56^.

#### Effect of metal ions on the protease activity

The effect of different metal ions on the activity of the protease was investigated by measuring the residual activity in the presence of 5 and 10 mM of each metal ion. As shown in Fig. 8c, Na^+^, K^+^, Ba^2+^, CO^2+^, Ca^2+^, and Mg^2+^ increased enzyme activity by 10–30% at most, while Hg^2+^, Pb^2+^, and Zn^2+^ inhibited the enzyme activity by 40–60% compared to the control. Among all metal ions, KCl induced the most significant effects (*p* < 0.0001) on enzyme activity. A similar increase in protease activity from *Aeromonas caviae* NRRL B-966 due to Mg^2+^ and Ca^2+^ was observed by Karunakaran and Devi^62^. In contrast, it has been reported that Zn ^2+^, Ca ^2+^, and Mg ^2+^ ions decreased the protease activity of *Aeromonas hydrophila* Ni 39. This indicates that the influence of metal ions on protease activity differs from one enzyme to another^59^. Different bacterial strains that inhabit various environments may require different metal ions to activate their enzymes^58^.

#### Effect of chemical modification and inhibitors on the protease activity

**Fig. 9:**
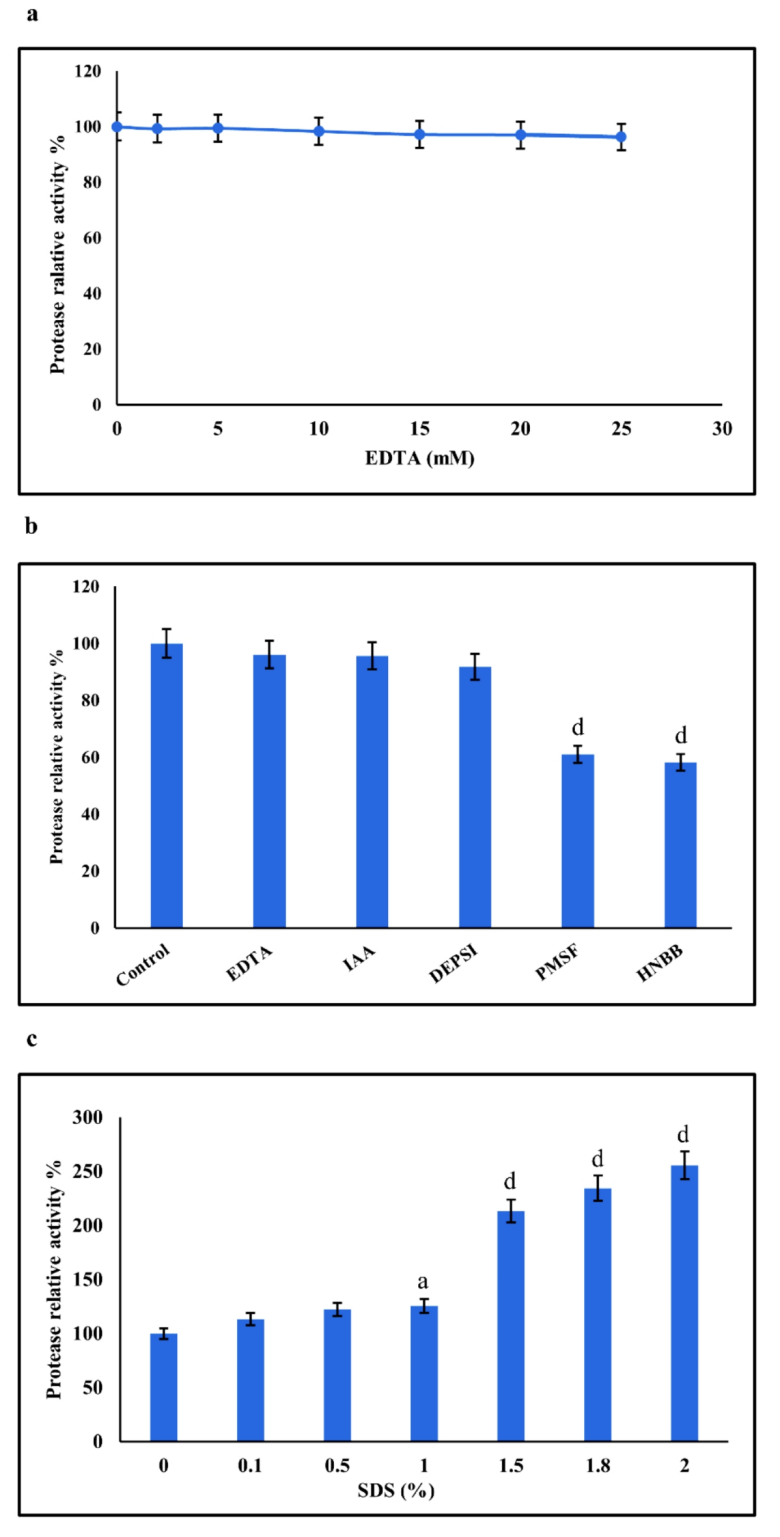
Effect of EDTA concentrations on protease activity produced by *Aeromonas* spp. (**a**). Effect of various enzyme inhibitors on the activity of the protease produced by *Aeromonas* spp. (**b**). Effect of SDS concentrations on protease activity produced by *Aeromonas* spp. (**c**). Data are presented as mean ± SD, and (**a**, **b**, **c** and **d**) represent significant differences from the control at *p* < 0.05, 0.01, 0.001, and 0.0001, respectively.

The investigation into the effect of EDTA concentrations on protease activity showed that the enzyme activity was not inhibited by EDTA, suggesting that the enzyme was resistant to metal ion chelation and was not a *metallo*-type of enzyme (Fig. 9a)^63^. This indicates the presence of a metal ion-independent type of enzyme^64^. Additionally, the impact of various protease inhibitors was explored to determine the nature of the protease. As demonstrated in Fig. 9b, both PMSF and HNBB significantly reduced protease activity by 39% and 42% respectively, compared to the control (untreated) (*p <* 0.0001). Other protease inhibitors such as IAA, DEPSI, and EDTA did not significantly affect enzyme activity. It can be suggested that the extracellular protease from *Aeromonas* spp. may be a serine protease, as indicated by the significant reduction in its catalytic ability due to the classical serine protease inhibitor, PMSF^65^. In the case of HNBB, which inhibits the tryptophan residue at the active sites of enzymes^66^, it is demonstrated that the amino acid tryptophan is present in the active site or structure of the enzyme, and its modification can reduce protease activity. A similar result was obtained with the *Aeromonas hydrophila* Ni 39 protease, which was resistant to EDTA but inhibited by PMSF by approximately 90%^59^.

#### Effect of SDS on the protease activity

The effect of surfactants on the activity of the partially purified protease was investigated by measuring the residual activity in the presence of varying concentrations of SDS. The results, as illustrated in Fig. 9c, revealed a significant increase in protease activity with rising concentrations of SDS, reaching up to 250% at a 2% SDS concentration. This increase suggests that the protease remains stable in the presence of SDS and can serve as a valuable additive in detergent formulations. Since SDS is known to denature proteins, this indicates that as the concentration of SDS increases, more casein is denatured, thereby exposing more substrate to the enzyme. Divakar et al. reported that the protease from *Aeromonas veronii* PG01 retained 90% and 80% of its relative activity in the presence of 0.25% and 0.5% SDS, respectively^67^. Another study revealed that the protease activity of *Aeromonas hydrophila* MSB16 decreased by only 3% in the presence of SDS^68^.

## Conclusion

In this study, the isolated bacterium (*Aeromonas* spp.) from the wastewater environment demonstrated that nature harbors suitable sources of various enzyme-producing bacteria for different purposes. The characteristics of the identified enzyme, such as its activity across a wide range of pH and temperature, as well as its resistance to numerous inhibitors and detergents, enhance its attractiveness for further studies. Based on preliminary studies, this enzyme has a robust structure and may serve as a suitable model for genetic investigations and protein studies. Our future research aims to assess the extracellular protease for its commercial potential and evaluate its application in the detergent industry. Such studies can be valuable for establishing a collection of enzyme-producing bacteria.

## Electronic supplementary material

Below is the link to the electronic supplementary material.

Supplementary Material 1

Supplementary Material 2

Supplementary Material 3

## Data Availability

The DNA-Seq data have been deposited in the National Center for Biotechnology Information (NCBI) database, under the accession number PP542514.1, and are accessible via the following URL: https://www.ncbi.nlm.nih.gov/nuccore/PP542514.1/.
