# Supplementary material for: A novel alkali and thermotolerant protease from Aeromonas spp. retrieved from wastewater
Source: Sci Rep. 2024 Oct 29;14:26000. doi: 10.1038/s41598-024-76004-w (PMC11522669; doi:10.1038/s41598-024-76004-w)
Supplement: Supplementary file 1 — Supplementary Material 1 [file 41598_2024_76004_MOESM1_ESM.pdf]

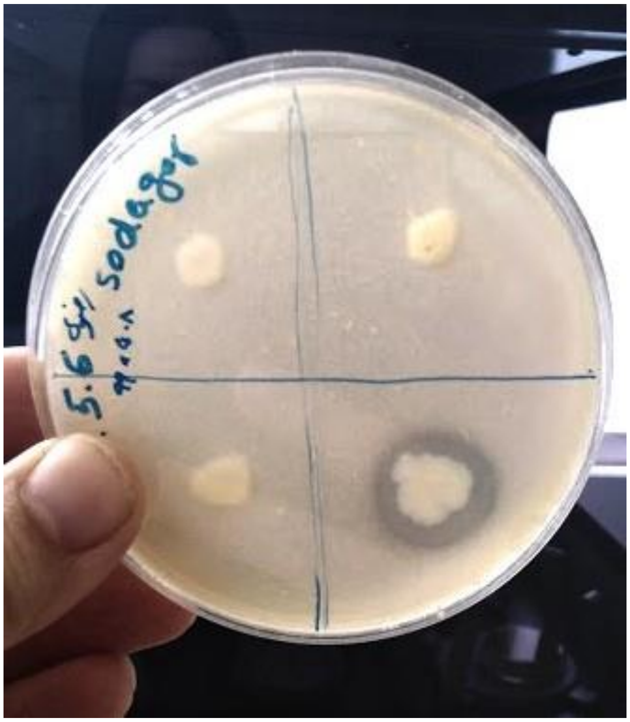

Figure. S1. Casein plate assay. The complete figure of the casein agar plate related to Figure 1.

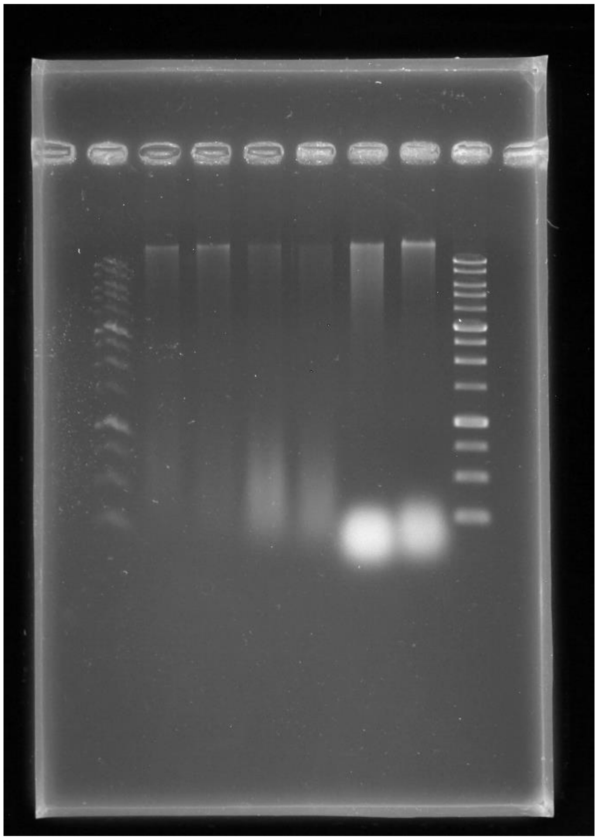

Figure. S2. Agarose gel of the extracted DNA related to Figure 3a.

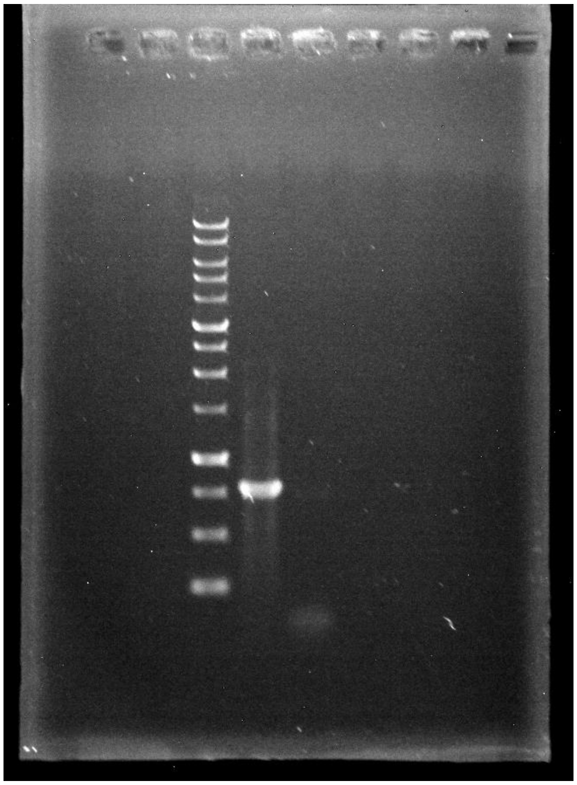

Figure. S3. Gel electrophoresis of PCR products using Eub_1 F and Eub_2 R primers related to Figure 3b.

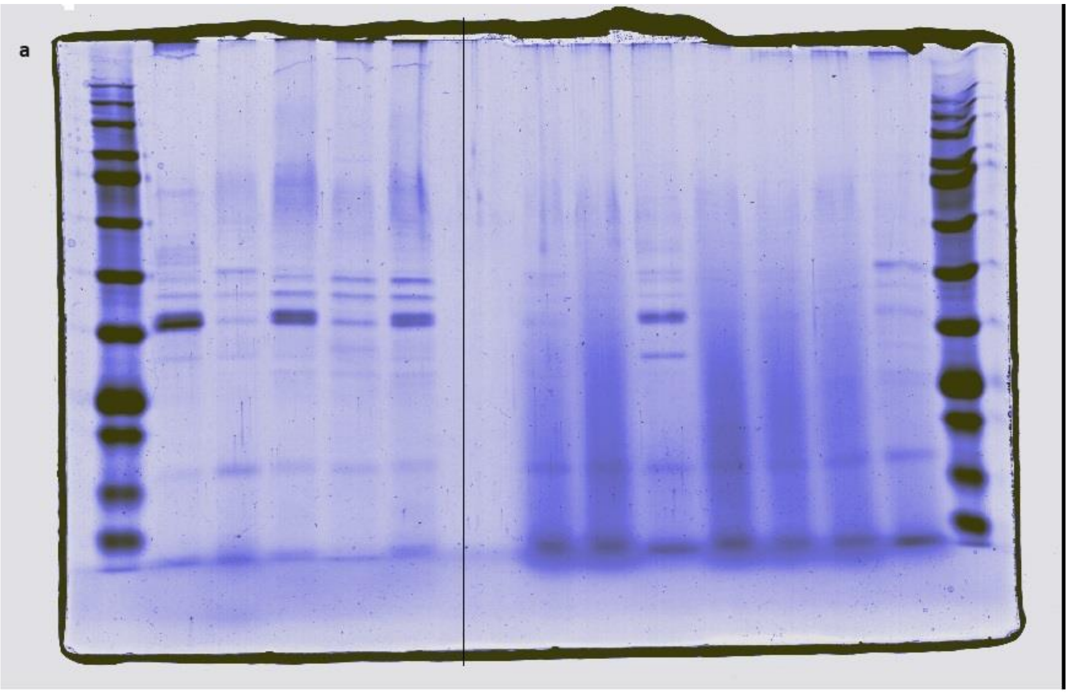

Figure. S4. SDS-PAGE gels (S4a, S4b) of protein related to different nitrogen sources as shown in Figure 5b. The black lines indicate the photo cut locations.

Figure. S4a

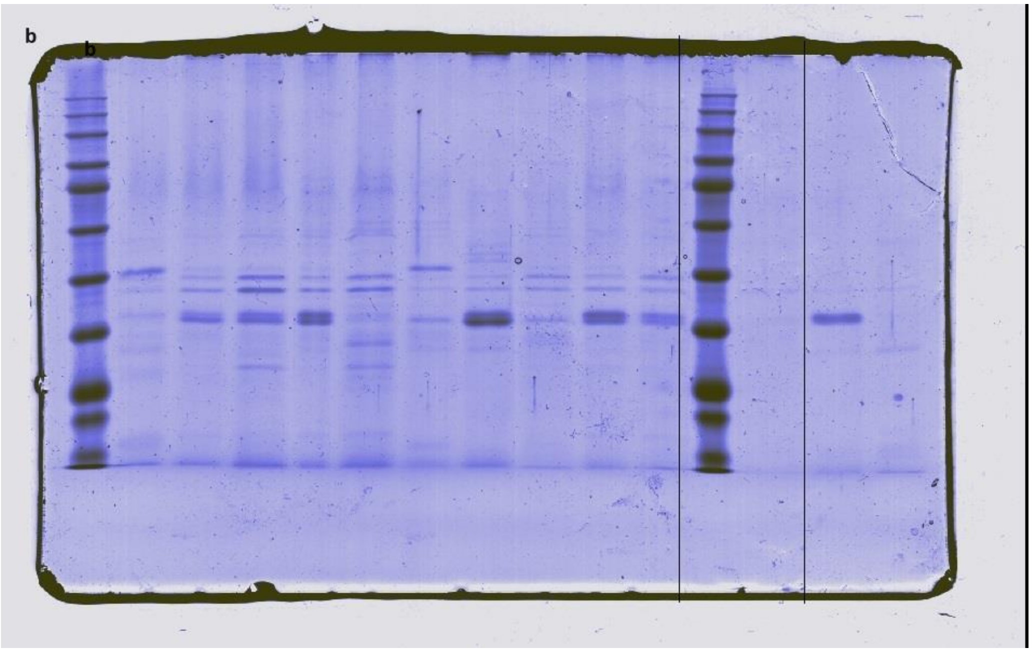

Figure. S4b

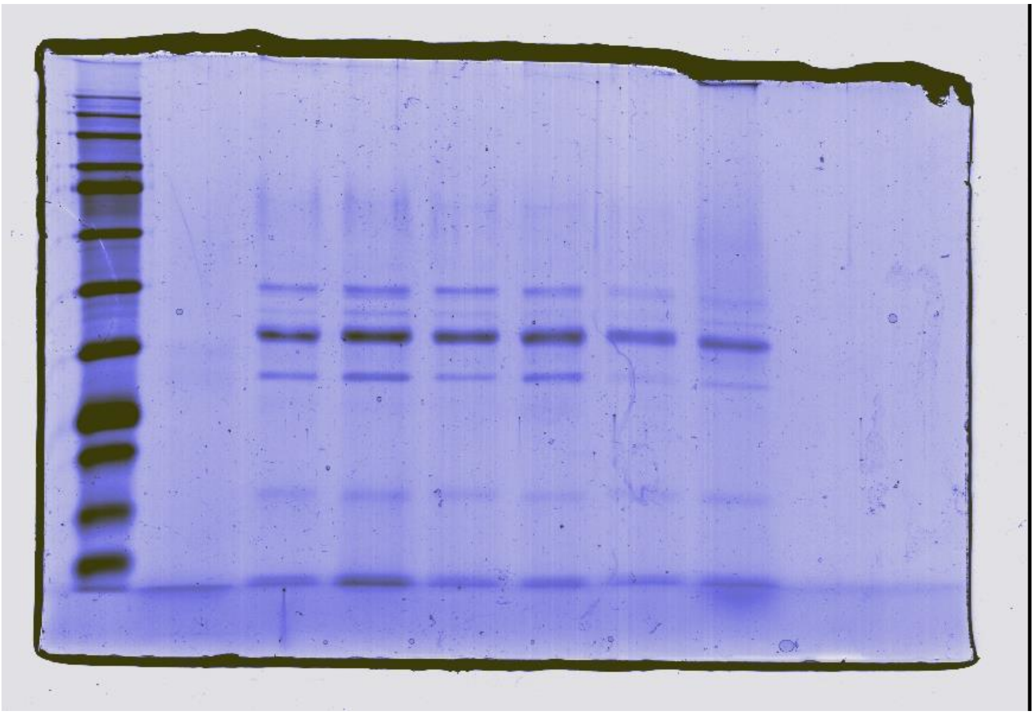

Figure. S5. SDS-PAGE gel of protein corresponding to different initial pH levels, as shown in Figure 6b.

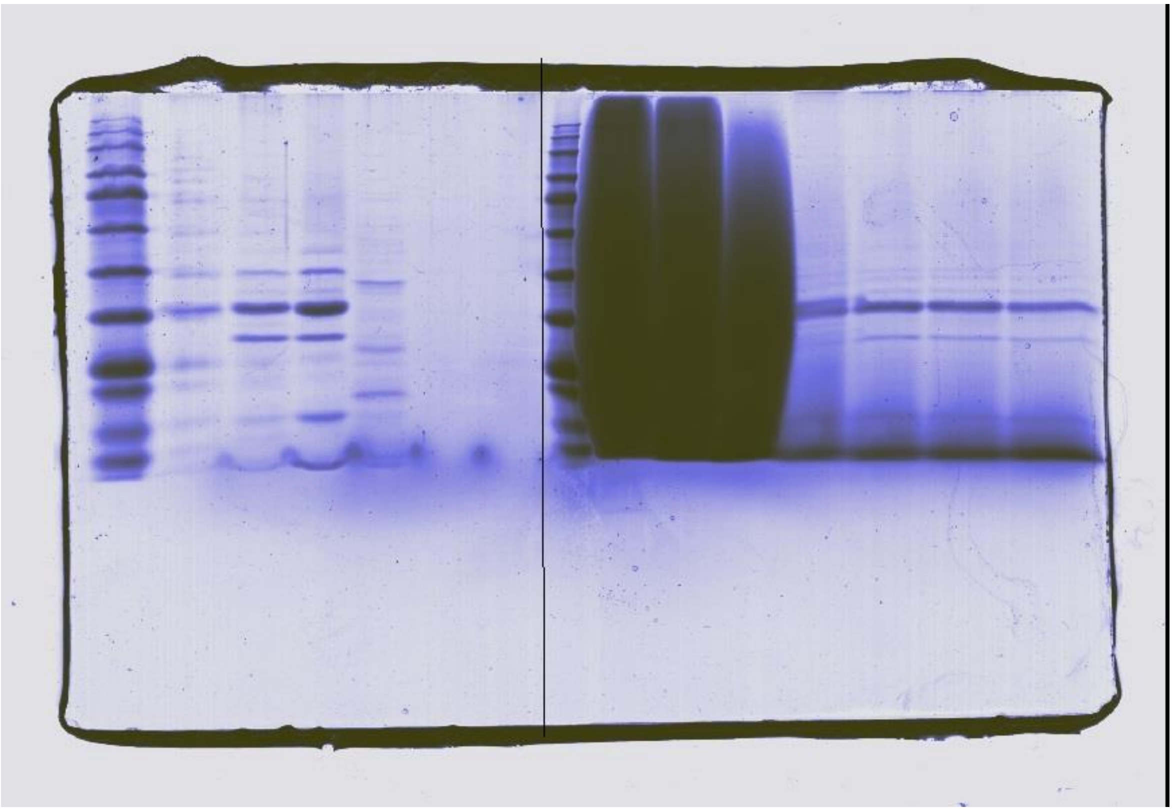

Figure. S6. SDS-PAGE gel of protein corresponding to different temperatures, as shown in Figure 6d. The black line indicates the photo cut location.

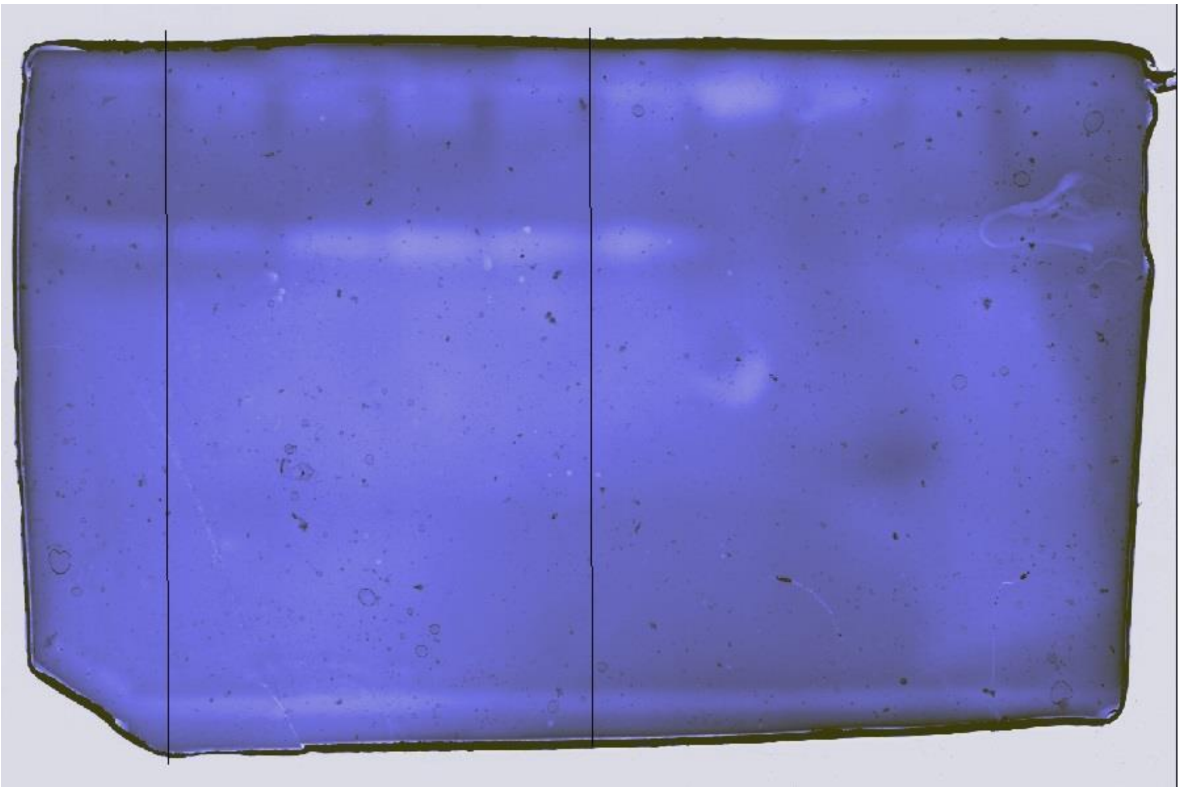

Figure. S7. 1% agarose gel related to zymography analysis, as shown in Figure 8a. The black lines indicate the photo cut locations.

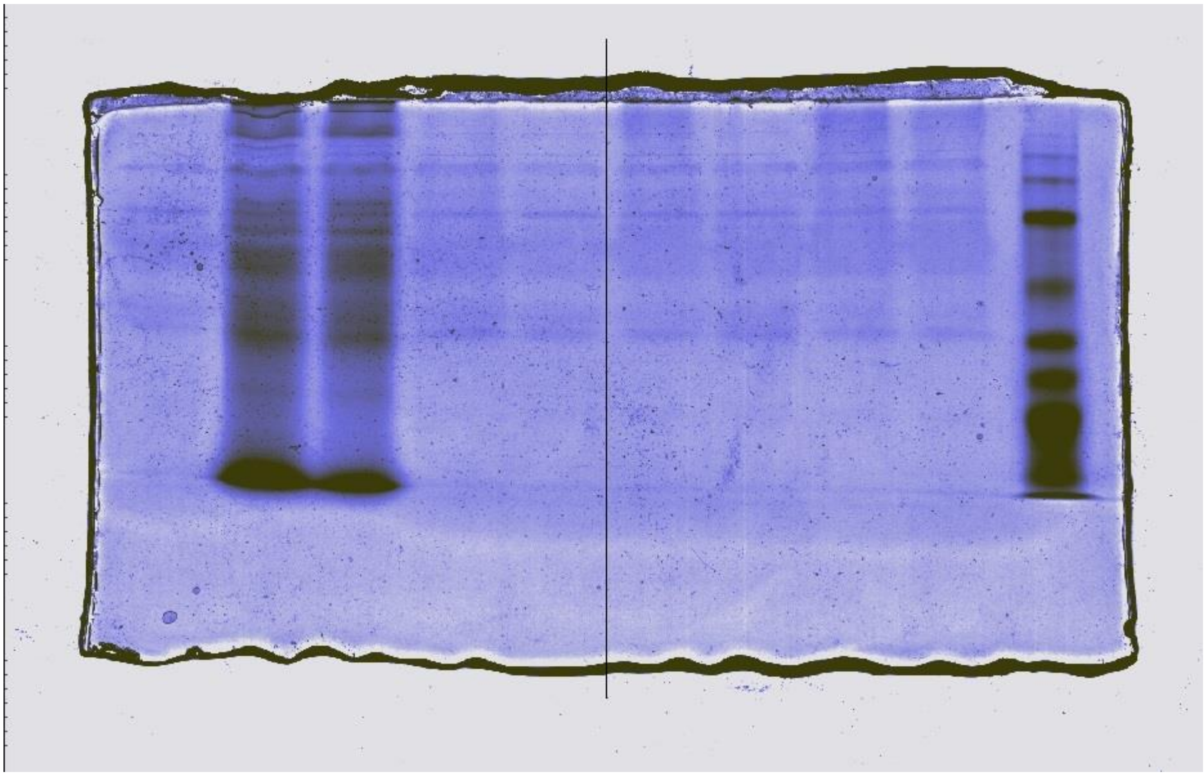

Figure. S8. 10% native polyacrylamide gel related to zymography analysis, as shown in Figure 8b. The black line indicates the photo cut location.
