# Supplementary material for: A novel alkali and thermotolerant protease from Aeromonas spp. retrieved from wastewater
Source: Sci Rep. 2024 Oct 29;14:26000. doi: 10.1038/s41598-024-76004-w (PMC11522669; doi:10.1038/s41598-024-76004-w)
Supplement: Supplementary file 3 — Supplementary Material 3 [file 41598_2024_76004_MOESM3_ESM.pdf]

Table S1. Clearing zone index (CI) for protease production by the bacterial colonies on a casein agar plate.

Colony. No	Halo zone + Colony diameter (cm)	Colony diameter (cm)	Clearing zone index
1	0.7±0.02	0.6±0.03	1.17
2	0.7±0.03	0.55±0.02	1.27
3	0.6±0.03	0.55±0.02	1.09
4	1.3±0.01	0.8±0.04	1.62

Data presented are average values ± SD of n = 3 experiments

Table S2. Morphological and biochemical identification of the isolate.

Morphological tests	Results
Gram's staining	Negative
Sporulation	Negative
Pigmentation	Creamy
Form	Irregular
Cell shape	Rod
Margin	Lobate
**Biochemical tests**	
Catalase	+
Oxidase	+
VP	+
Indole	+
Mannitol	-
Glucose	-
Lysine	+
H_2S	+
Xylose	-
Urease	-
Citrate	+
Ornithine	-
TDA	-
ONPG	-
OF Test	Facultative Anaerobes

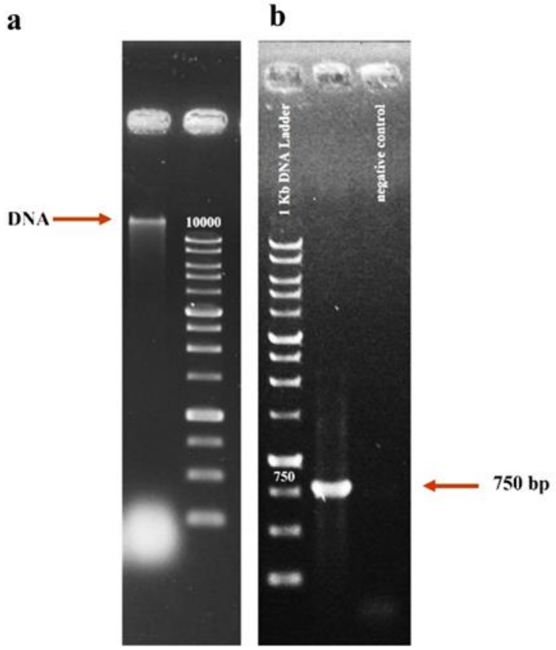

Figure S1. Agarose gel of the extracted DNA (cropped) (a). Gel electrophoresis of PCR products using Eub_1 F and Eub_2 R primers. The 750 bp amplified 16S rDNA fragment of the bacterium monitored on 1% agarose gel (cropped) (b). The original gels are presented in Supplementary file 1, Figs. S2 and S3 online.
